# A humanised anti-IGF-1R monoclonal antibody (AVE1642) enhances Bortezomib-induced apoptosis in myeloma cells lacking CD45

**DOI:** 10.1038/sj.bjc.6604839

**Published:** 2009-01-22

**Authors:** G Descamps, P Gomez-Bougie, C Venot, P Moreau, R Bataille, M Amiot

**Affiliations:** 1 Centre de recherches en Cancérologie Nantes/Angers, INSERM, UMR 892, UFR Médecine et Techniques Médicales, Université de Nantes, Nantes Atlantique Universités, Nantes, France; 2 Oncology Therapeutic Department, Sanofi-Aventis, 13 quai Jules Guesde, Vitry sur Seine 94403, France; 3 UFR Médecine et techniques Médicales, Université de Nantes, Hématologie Clinique, CHU de Nantes, Nantes, France

**Keywords:** multiple myeloma, IGF-1/IGF-1R, bortezomib, CD45, translocation (4,14)

## Abstract

The humanised form of an antagonistic anti-IGF-1R mAb (AVE1642) selectively inhibits the growth of CD45^neg^ myeloma cells. AVE1642 strongly increased bortezomib-induced apoptosis, correlated with an increase of Noxa expression. These results support the therapeutic use of anti-IGF-1R/bortezomib in CD45^neg^ Myeloma patients, particularly those with the most aggressive form, *t*(4,14).

The therapeutic use of monoclonal antibodies (mAb) has emerged as a very potent clinical strategy in the treatment of cancer (Houshmand and Zlotnik, 2003; Hudis, 2007). In multiple myeloma (MM), we previously demonstrated that an antagonistic anti-IGF-1R mAb (EM164, also named mAVE1642) can selectively inhibit the growth of CD45^neg^ human myeloma cell lines (HMCLs) (Descamps *et al*, 2006). MM is an incurable plasma-cell malignancy. The subgroup of MM patients lacking CD45 is associated with less favourable clinical outcomes that require new treatments (Moreau *et al*, 2004). Furthermore, it was shown that HMCL and MM patients with either *t*(4,14) or *t*(14,16) chromosomal translocations expressed a higher level of IGF-1R (Bataille *et al*, 2005). We previously demonstrated that EM164 selectively inhibits IGF-1R but not insulin signalling in myeloma cells (Descamps *et al*, 2006). Thus, EM164 displays a very potent and specific effect of IGF-1R downregulation and complete blockade of IGF1-induced Akt and Erk phosphorylation. Similar results with AVE1642 recently confirmed using the fully human anti-IGF-1R antibody named A12 (Wu *et al*, 2007). Furthermore, it was shown that the A12 antibody can reduce angiogenesis as it inhibits the *in vitro* secretion of VEGF in HMCL (Wu *et al*, 2007). Altogether, these studies strongly support the use of EM164 in combination regimens.

In addition to alkylating agents and corticosteroids, bortezomib, a highly selective and potent inhibitor of the 26S proteasome, has emerged as a novel class of potent agents in the treatment of MM (Richardson *et al*, 2006a). In this study, we investigated the effects of bortezomib and AVE1642 in combination on the induction of apoptosis in HMCLs. Our group previously reported that bortezomib-induced apoptosis in HMCLs was associated with Noxa (a BH3-only protein) upregulation (Gomez-Bougie *et al*, 2007). Therefore, in the present study, we also examined the impact on Noxa of the combination of bortezomib with AVE1642.

## Materials and methods

### Monoclonal antibodies and reagents

The anti-IGF-1R mAb AVE1642 was kindly provided by Sanofi-Aventis/Immunogen (Vitry sur Seine, France). r-IL-6 was from Novartis (Basel, Switzerland). The mAb against Noxa was from Alexis Biochemicals (Nottingham, UK). The antibody against caspase-3 (mouse mAb) was from Santa Cruz Biotechnology (Tebu-Bio, Le Perray en Yvelines, France). Anti-actin mAb was purchased from Chemicon International (Temecula, CA, USA).

### Cell culture conditions

We used LP-1, NCI-H929 and JIM-3 human myeloma cell lines and the IL-6-dependent human myeloma cell lines XG-1, XG-6, BCN and NAN-3 HMCL. Cell lines were grown as described earlier (Descamps *et al*, 2006). The characteristics and phenotypes of these HMCLs have been recently reviewed by Bataille *et al*, 2006.

### Cell viability and detection of apoptotic cells

Cell viability was determined by vital dye (0.4%. eosin) exclusion and assessed by visual inspection in a haemocytometer. Cell death was assessed by Apo 2.7 staining (Beckman Coulter, Roissy, France) or by annexin V staining (Beckman Coulter) according to the manufacturer's recommendation. Flow cytometry analysis was performed on a FACSCalibur using the Cell Quest software (Becton Dickinson, San Jose, CA, USA).

### Proliferation assays

Myeloma cells (10^4^ cells per well) were cultured in triplicate in 96-well plates for 72 h. Cells were pulsed with 1 *μ*Ci [^3^H] thymidine during the last 8 hours of culture, harvested onto glass filters with an automatic cell harvester (Perkin Elmer, Courtaboeuf, France), and the uptake of [^3^H] thymidine was monitored using a 1450-Microbeta Jet *β*-counter (Perkin Elmer).

### Protein extraction and western blot analysis

Western blot analysis of total cell lysates was performed according to published protocols (Descamps *et al*, 2006). Immunoblots were done using anti-Noxa, anti-caspase-3 and anti-actin antibodies.

### Statistical analysis

For statistical analysis, we used the non-parametric Spearman's rank test and the sign test.

## Results

**Figure 1:**
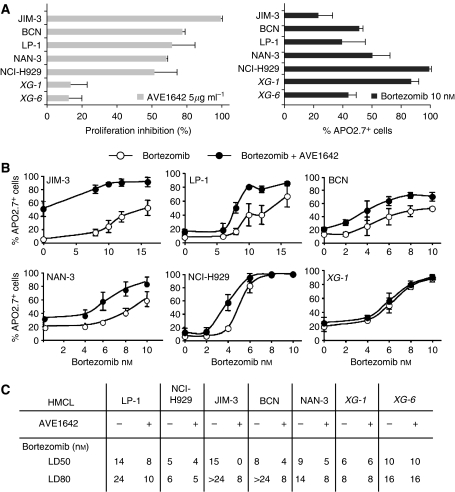
(**A**) Sensitivity of HMCLs to AVE1642 and bortezomib. Myeloma cells were cultured in the presence or absence of AVE1642 (5 *μ*g ml^−1^) for 3 days. For the IL-6-dependent HMCLs (XG-1, XG-6, BCN and NAN-3) 3 ng ml^−1^ of IL-6 was added. Results are shown as mean (±s.d.) of percent proliferation inhibition by [^3^H] thymidine incorporation of triplicate cultures, compared with the untreated control. For Bortezomib sensitivity, cells were treated with 10 nM of bortezomib for 48 h in culture conditions. Then, cell death was assessed by Apo 2.7 staining.(**B**) Bortezomib in association with AVE1642 is more efficiently able to induce apoptosis of CD45^neg^ HMCLs than bortezomib alone. Myeloma cells were pre-incubated in RPMI-1640 with 5% FCS in the absence or presence of AVE1642 (5 *μ*g ml^−1^) for 24 h. For the IL-6-dependent HMCLs (BCN, NAN-3, XG-1 and XG-6), 3 ng ml^−1^ of IL-6 is added. Then, cells were treated with different concentrations of bortezomib in the absence or presence of AVE1642 (5 *μ*g ml^−1^) for an additional 48 h. Cells were stained with Apo2.7-PE. (**C**) Bortezomib LD50 and LD80 in the presence or not of AVE1642. Myeloma cells were treated as above and LD50 and LD80 were determined.

In the present study, we used AVE1642, the humanised counterpart of EM164 that displays the same selectivity to inhibit the IGF-1 signalling but has no effect on insulin pathway as demonstrated earlier (Descamps *et al*, 2006). We have previously shown that EM164 strongly inhibits the growth of CD45^neg^ HMCLs, leading to a G1 growth arrest, whereas it has almost no effect on CD45^pos^ HMCL growth (Descamps *et al*, 2006). In this study, we demonstrated that AVE1642 retains this selective activity toward CD45^neg^ HMCLs. After 4 days of incubation with 5 *μ*g ml^−1^ of AVE1642, we observed growth inhibition ranging from 60 to 99% for the five CD45^neg^ HMCLs (BCN, NAN-3, LP1, NCI-H929 and JIM-3). In contrast, a very weak growth inhibition was observed for XG-1 and XG-6 CD45^pos^ HMCLs, at 13±10% and 12±10%, respectively (Figure 1A). Interestingly, we observed that the CD45^neg^ HMCLs had either a *t*(4,14) (*n*=4) or *t*(14,16) chromosomal translocation (*n*=1). We found that AVE1642 treatment did not induce any apoptosis in HMCLs, measured both by Apo2.7 and annexin staining, regardless of CD45 status, with the exception of JIM-3, where AVE1642 induced 50±4% of apoptotic cells (Figure 1B). Of note, JIM-3 has been reported to express very high IGF-1R levels (Descamps *et al*, 2006), which may explain its survival addiction to this target. Bortezomib has been shown to induce apoptosis in MM. As shown earlier (Gomez-Bougie *et al*, 2007), measurement of bortezomib activity using Apo2.7 staining confirmed that HMCLs exhibit different sensitivities to bortezomib treatment. HMCLs can be classified as follows: high sensitivity (NCI-H929, XG-1), intermediate sensitivity (NAN-3, BCN and XG-6) and weak sensitivity (LP1 and JIM-3). Of interest, our results show a strong statistical inverse correlation between bortezomib apoptosis induction and AVE1642 growth inhibition in this fairly large panel of HMCLs (*P*=0.042, Spearman's test). We then tested whether AVE1642 was able to enhance bortezomib-induced apoptosis. AVE1642 significantly enhanced bortezomib-induced apoptosis in all CD45^neg^ HMCLs (Figure 1B). Moreover, a pretreatment with AVE1642 for 24 h was more efficient than simultaneous treatment with AVE1642/bortezomib (data not shown). Increased apoptosis induction due to the AVE1642/bortezomib combination was independent of the bortezomib sensitivity status of HMCL, but was significantly correlated with the AVE1642 sensitivity (*P*=0.038). As expected, the AVE1642/bortezomib combination did not modify bortezomib-induced apoptosis in CD45^pos^ HMCLs. After determination of the bortezomib LD50 and LD80 (bortezomib concentrations where 50 and 80% of cells are stained with Apo2.7, respectively), we measured the impact of AVE1642 addition on those values. In CD45^neg^ HMCLs, but not in CD45^pos^ HMCLs, addition of AVE1642 induced significant decreases in the bortezomib LD50 and LD80 values. More specifically, Figure 1C shows that bortezomib LD80 values were statistically shifted from >24 to 14 nM to less than 10 nM (*P*<0.05). Altogether, these results demonstrate synergy between bortezomib and AVE1642 in CD45^neg^ HMCLs.

**Figure 2:**
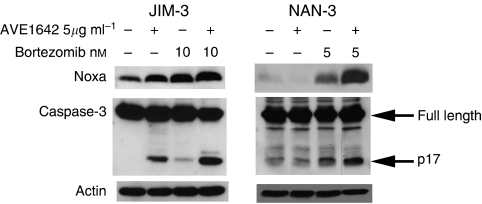
Combination of AVE1642 with bortezomib induces Noxa induction, and caspase-3 activation in CD45^neg^ HMCLs. HMCLs were treated for 48 h with the indicated dose of Bortezomib in the presence or absence of AVE1642 (5 *μ*g ml^−1^). Cells were lysed, subjected to SDS–PAGE, transferred to PDVF membrane and probed with the indicated antibody. Protein loading was controlled with anti-actin.

Our group has recently pinpointed mechanisms by which bortezomib promotes apoptosis, showing that Noxa induction is linked to bortezomib-induced apoptosis (Gomez-Bougie *et al*, 2007). BH3-only Noxa is a sensitiser molecule that induces the release of BH3-only activators that are responsible for Bax activation and activation of the mitochondrial apoptotic pathway (Letai *et al*, 2002). In NAN-3 HMCLs, we confirmed that bortezomib-induced apoptosis was associated with Noxa upregulation. Figure 2 shows that this upregulation was strongly increased by the addition of AVE1642 to CD45^neg^ HMCLs, whereas this upregulation was not detected in CD45^pos^ HMCLs (result not shown).

In JIM3 HMCLs, a moderate Noxa protein increase was also observed by AVE1642 alone; however, the combination of AVE1642 with bortezomib led to stronger Noxa upregulation. Noxa upregulation was associated with caspase-dependent cell death, as shown by caspase3 activation (detected by p17 cleaved fragment). Moreover, in JIM-3 HMCLs, a significant cleavage of caspase3 was observed with AVE1642 alone, coinciding with moderate apoptosis and Noxa induction (Figure 2).

## Discussion

This study demonstrates synergy between bortezomib and AVE1642 for apoptosis induction in CD45^neg^ HMCLs. Indeed, we have shown earlier that the presence of the CD45 phosphatase inhibits IGF-1 signalling by a direct interaction between CD45 and IGF-1R, which probably dephosphorylates IGF-1R and results in an inhibition of IGF-1 signalling. This may explain the restricted effect of the combination of bortezomib and AVE1642 on CD45^neg^ HMCLs. Of note, CD45^neg^ HMCLs specifically include HMCLs with the most aggressive 14q32 translocations (*t*(4,14) and *t*(14,16)), and these also present higher levels of IGF-1R expression (Bataille *et al*, 2005). Altogether, we showed that the combination of bortezomib and AVE1642 may be particularly an efficient strategy for CD45^neg^ HMCLs having the translocations *t*(4,14) and *t*(14,16) and thus expressing the highest IGF-1R levels. Furthermore, our CD45^neg^ HMCL panel shows that the increased potency of bortezomib when combined with AVE1642 is directly related to the sensitivity of the individual cell line to AVE1642. Finally, we show that AVE1642 strongly increases bortezomib-induced apoptosis in correlation with an increase of both Noxa expression and caspase-3 activation. Furthermore, Noxa induction levels were correlated with the increased apoptotic effect of the AVE1642/bortezomib combination. This result emphasises the importance of Noxa as a sensitiser molecule that is able to induce the release of the BH3-only activators that are responsible for Bax activation.

Taken together, these results suggest that the combination of anti-IGF-1R therapies with bortezomib treatment could be beneficial for MM patients and may be particularly useful for the subpopulation of patients lacking CD45. Of note, this association could provide significant improvements in the treatment of the most aggressive and resistant forms of MM, those with *t*(4,14). These forms of MM are characterised by both the lack of CD45 and a high expression of IGF-1R (Bataille *et al* 2005). In addition, this treatment strategy may help to improve the management of bortezomib dosage in a clinical setting as this agent has a very narrow therapeutic window and can induce severe peripheral sensory neuropathy (Richardson *et al*, 2006b).
